# In Vitro Validation of Pulsed Electromagnetic Field (PEMF) as an Effective Countermeasure Against Inflammatory‐Mediated Intervertebral Disc Degeneration

**DOI:** 10.1002/jsp2.70077

**Published:** 2025-05-19

**Authors:** Laura Guarnaccia, Laura Begani, Silvana Pileggi, Mauro Pluderi, Stefano Borsa, Claudia Fanizzi, Massimiliano Domenico Rizzaro, Giorgio Fiore, Laura Fontana, Rolando Campanella, Chiara Cordiglieri, Chiara Gaudino, Giovanni A. Alotta, Monica Miozzo, Emanuele Garzia, Emanuela Barilla, Lorenzo Fassina, Laura Riboni, Marco Locatelli, Giovanni Marfia, Stefania E. Navone

**Affiliations:** ^1^ Center of Aerospace Medicine for Advanced Therapies (CeMATA), Laboratory of Experimental Neurosurgery and Cell Therapy, Neurosurgery Unit Foundation IRCCS ca' Granda Ospedale Maggiore Policlinico Milan Italy; ^2^ Andremacon Srl Milan Italy; ^3^ Neurosurgery Unit Foundation IRCCS Ca' Granda Ospedale Maggiore Policlinico Milan Italy; ^4^ Medical Genetics Unit ASST Santi Paolo e Carlo Milan Italy; ^5^ Istituto Nazionale Genetica Molecolare Romeo Ed Enrica Invernizzi Milan Italy; ^6^ Department of Neuroradiology Azienda Ospedaliero‐Universitaria Policlinico Umberto I Rome Italy; ^7^ Aerospace Medicine Institute “A. Mosso” Italian Air Force Milan Italy; ^8^ Reproductive Medicine Unit, Department of Mother and Child San Paolo Hospital Medical School, ASST Santi Paolo e Carlo Milan Italy; ^9^ Department of Electrical, Computer and Biomedical Engineering University of Pavia Pavia Italy; ^10^ Department of Medical‐Surgical Physiopathology and Transplantation University of Milan Milan Italy

**Keywords:** inflammation, intervertebral disc degeneration, microglia, pilots, pulsed electromagnetic field, space

## Abstract

**Background:**

Intervertebral disc (IVD) degeneration (IDD) is the main contributor to chronic low back pain (LBP), the leading cause of disability worldwide, with a significant impact on the quality of life and health of common people. The etiology of IDD is still unclear, but it has been largely demonstrated the crucial role of inflammation and neuroinflammation in the pathological and degenerative cascade of events characterizing IVD degeneration.

**Aim:**

In this study, we evaluated the potential therapeutic effect of pulsed electromagnetic field (PEMF) on human degenerated IVD (D‐IVD) cells collected from patients who underwent discectomy.

**Materials & Methods:**

The experimental plan to test our hypothesis, involved viability assay, reactive oxide species/nitrite production, gene, and protein expression. To recapitulate the pro‐inflammatory disc microenvironment occurring during IDD, interleukin‐1β (IL‐1β) was administered to IVD cell culture. Then, to dissect the contribution of neuroinflammatory condition to immune component, microglial cells were co‐cultured with IVD‐conditioned media, and viability and expression of inflammatory markers were detected.

**Results:**

Our data prove that in the IVD degenerative microenvironment, the increase of pro‐inflammatory mediators, extracellular matrix degradative enzymes, and neuroinflammatory markers could be reduced by PEMF therapy, resulting in an overall improvement of degenerative condition and LBP.

**Conclusion:**

These results represent an impactful novelty for the management of people suffering from LPB, in terms of symptom relief and reduction of social‐health system burden.

## 1 Introduction

Low back pain (LBP) represents a serious socio‐economic burden affecting people of all ages and represents one of the three main causes of disability in developed countries [1]. Although LBP etiology is still not completely understood, a well‐established contributor is intervertebral disc (IVD) degeneration (IDD) (Risbud and Shapiro [2]).

IDD is a pathological process that occurs because of the normal aging process; nevertheless, it can be considered a complex disorder associated with multiple risk factors, including genetic susceptibility, gender, mechanical loading, smoking, and vibration [3, 4].

Nowadays, current therapies for IDD include conservative treatments (administration of anti‐inflammatory drugs) and, with the aggravation of symptoms, surgical treatments to symptomatic back pain. However, there is a significant number of patients in which these conventional therapies often fail, which is why new treatment options including physical therapy, tissue engineering, growth factor therapy, gene therapy, and cell‐based treatments are continuously under investigation [5, 6].

Microscopically, IDD is characterized by a sustained overproduction of pro‐inflammatory cytokines and chemokines causing matrix degeneration, cell senescence, and apoptosis, nerve and vascular ingrowth, and eventually pain. Several mediators, such as inteleukin‐1β (IL‐1β) secreted by resident IVD cells and circulating immune cells that infiltrate within the degenerating IVD, contribute to exacerbate the detrimental IVD microenvironment [7, 8]. In particular, we previously observed that the IDD inflammatory microenvironment promotes the activation and proliferation of neuroglia [9], which, in turn, reinforces the cytokine‐mediated degenerative process, resulting in the amplification of the inflammatory cascade and the worsening of the overall degenerative condition (Risbud and Shapiro 2013).

Once switched on, inflammation initiates a detrimental feedback loop into IVD, with recruited immune cells producing chemokines and cytokines (IL6, IL‐1β, TNF‐α) that upregulate the expression of matrix degradative enzymes, such as metalloproteinases (MMPs) and A Disintegrin and Metalloproteinase with Thrombospondin motifs (ADAMTS) (Risbud and Shapiro 2013).

Further, the biochemical changes occurring during the early stages of IDD also implicate the breakdown of proteoglycans, with the gradual modification of IVD collagen types. Among them, aggrecan represents the major proteoglycan of IVD structures and it contributes to the swelling pressure responsible for the resistance to compressive loading [10]. Of relevance, the loss of hydration that a degenerating IVD experiences is particularly due to the aggrecan impairment because of the decrease of aggrecan hydrophilic and water‐retaining chondroitin sulfates.

Scientific literature and clinical data suggest the urgency to explore novel therapeutic and preventive approaches that could robustly affect the quality of life of patients suffering from LBP, as well as healthcare costs.

Of interest, recent studies reported increasing evidence for IDD in pilots and astronauts, currently exposed to peculiar conditions. In space, in fact, the lack of gravity leads to pathophysiological modifications in different districts of the organism, as well as the musculoskeletal system, with the consequent effect on health and performance of space crews [11]. Under a weightless environment, as during a spaceflight, the compressive force exerted against IVD significantly decreases, thus resulting in a reduced secretion of proteoglycan and, consequently, in the alteration of matrix components [12]. This, in turn, breaks the homeostasis of IVD and promotes its progressive degeneration. From this evidence, it appears reasonable that discovering new etio‐pathological associations and novel routes of treatment may have a dual translational relevance, affecting also aerospace medicine and military staff health management [11].

In the present study, we focused on the potential use of pulsed electromagnetic fields (PEMF), a non‐invasive form of physical treatment, which resulted to be promising in the treatment of various musculoskeletal and inflammatory‐related diseases because of its anti‐inflammatory and analgesic effects as well as its capability to accelerate bone repair [13, 14]. Currently, the efficacy of PEMF therapy in the treatment of neuroinflammation in IDD remains unexplored.

Considering all these premises, here we tested the effect of PEMF on human degenerated IVD (D‐IVD) cells collected from patients who underwent discectomy. To recapitulate the pro‐inflammatory disc microenvironment occurring during IDD, IL‐1β was administered. In addition, microglial cells were co‐cultured with IVD conditioned media (CM) to mimic the neuroinflammatory condition, to test the potential beneficial effect of PEMF therapy.

Our results proved the ability of PEMF to counteract inflammation, matrix degradation, and consequently, to reduce pain mediators, resulting in an overall improvement of the degenerative condition.

## 2 Materials and Methods

### 2.1 Isolation of Intervertebral Degenerated Disc Cells

The study protocol was approved by our institutional ethics committee (DISCOLAB n. 232_2018bis) and informed consent to participate in the study was obtained from all the patients recruited. In our study, samples of degenerated IVD (D‐IVD) were collected from the lumbar column of 10 patients undergoing surgeries for lumbar discectomy (Table 1). From each patient, a lumbosacral MRI was collected. Nucleus pulposus (NP) was separated from the annulus fibrosus (AF) using a stereotaxic microscope, collected, and placed in a falcon with Dulbecco's Modified Eagle Medium (DMEM, Gibco, Grand Island, NY) supplemented with 1% of Penicillin–Streptomycin (Sigma–Aldrich, Basel, Switzerland), until sample digestion. NP samples were enzymatically digested with Trypsin (Gibco) for 4 h at 37°C instead of the commonly used Collagenase II: in previous works, it has been observed that tissue digestion performed with Trypsin was faster (4 h vs. 6 h with Collagenase II) (Basatvat et al. [15]) and, from our experience, yielded superior results in terms of cellular yield and cell viability, representing a valid alternative method. The digested tissues were filtered by a 70 μm cell strainer (Falcon, Becton Dickinson, Allschwil, Switzerland) to remove debris and obtain a cell suspension, centrifuged at 400 g for 10 min. Human degenerated disc cells (D‐IVD cells) derived from NP samples were seeded in 75 cm^2^ flasks (2 × 10^5^ cells/cm^2^) and incubated at 37°C, 5% CO_2_ in Basal Medium (CTRL) formulated as follows: RPMI (Sigma‐Aldrich) supplemented with 10% of Fetal Bovine Serum (FBS, Life Technologies, Carlsbad, CA, USA) and 1% of penicillin–Streptomycin (Sigma‐Aldrich). Notably, the experiments described below, if not otherwise specified, were performed on *n* = 10 biological replicates of D‐IVD cells, with each experiment run in triplicate.

**TABLE 1:** Clinical and demographic data of IVD donors.

	Age	Sex	Diagnosis	Surgery	IVD level	Grade
1	50	M	DLS	Lumbar discectomy	L3‐L4	4
2	52	F	DLS	Lumbar discectomy	L4‐L5	4
3	60	M	DLS	Lumbar discectomy	L4‐L5	4
4	55	F	DLS	Lumbar discectomy	L3‐L4	4
5	58	F	DLS	Lumbar discectomy	L4‐L5	4
6	59	M	DLS	Lumbar discectomy	L3‐L4	4
7	53	M	DLS	Lumbar discectomy	L4‐L5	4
8	25	M	DLS	Lumbar discectomy	L4‐L5	4
9	25	M	DLS	Lumbar discectomy	L4‐L5	4
10	48	F	DLS	Lumbar discectomy	L4‐L5	4

*Note:* Grade is referred to Pfirman grading system, based on radiological images.

Abbreviation: DLS, degenerative lumbar spondylolisthesis.

### 2.2 Microglial Cell Culture Isolation and Expansion

The N9 murine microglia (RRID:CVCL 0452) was cultured in Iscove's Modified Dulbecco's Medium (IMDM, Life Technologies) with 4 mM Glutamine, 25 mM HEPES (Gibco), 1% penicillin/streptomycin solution (Sigma‐Aldrich) and supplemented with 5% FBS (Life Technologies). Cells were grown at 37°C in a humidified incubator in 95% air‐5% CO_2_ and passaged every 3–4 days using Tryple Select (Gibco), at a seeding concentration of 4 × 10^3^ cells/cm^2^.

### 2.3 Pulsed Electromagnetic Field (PEMF) Treatment

**FIGURE 1:**
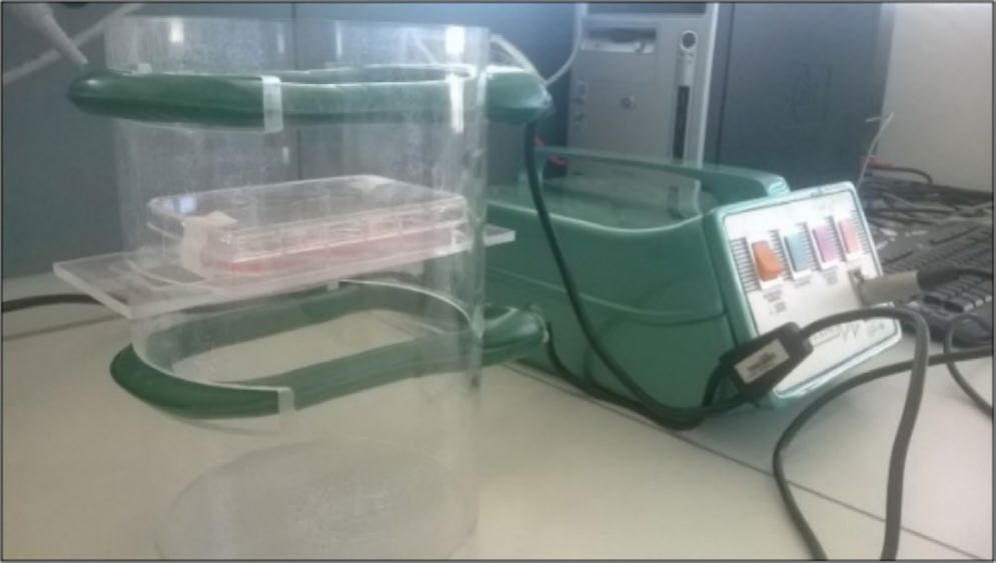
Electromagnetic Bioreactor. The device was used to generate a pulsed electromagnetic field in human IVD cells. The system was composed of a windowed tube carrying a well plate and two solenoids (Helmholtz coils) powered by a Biostim SPT pulse generator. The electromagnetic protocol had the following characteristics: Magnetic field intensity equal to 2 ± 0.2 mT, induced electric tension equal to 5 ± 1 mV, signal frequency of 75 ± 2 Hz, and pulse duration of ∼1.3 msec.

The electromagnetic bioreactor (a generator of pulsed electromagnetic field, PEMF) consisted of a carrying structure custom‐designed in a tube of polymethylmethacrylate: the windowed tube carried a well plate and two solenoids (Helmholtz coils) powered by a Biostim SPT pulse generator (IGEA Medical, Carpi, Italy). The IGEA PEMF device delivers a specific PEMF with the following characteristics: the signal is a pulse of 1.3 milliseconds duration with a frequency of 75 Hz; the solenoid induces a magnetic field of peak intensity of 2.0 ± 0.5 mT (Figure 1).

### 2.4 D‐IVD Cell Treatment

D‐IVD cells (1.2 × 10^5^ cells/cm^2^) were plated in *n* = 4 different experimental conditions: (i) CTRL, (ii) added with IL‐1β (10 ng/mL, ReliaTech GmbH, Wolfenbuttel, Germany) to reproduce the pro‐inflammatory degenerating microenvironment; (iii) under PEMF exposition, for 4 h every day for five consecutive days at room temperature (RT); (iv) IL‐1β + PEMF, stimulated with IL‐1β and exposed to PEMF for 4 h every day for five consecutive days at RT. Notably, D‐IVD cells in the CTRL and IL‐1β groups were maintained at RT for 4 h every day for 5 days to recapitulate the same environmental condition of cells exposed to PEMF. After the different treatments, cell culture media were collected and centrifuged at 300 g for 10 min and stored at −20°C for the subsequent experimental analyses, and herein referred to as conditioned medium (CM).

### 2.5 Flowcytometric Analysis (FACS)

For the immunophenotypic characterization, D‐IVD cells (1 × 10^5^/tube) isolated from NP of 10 human degenerated IVD samples, except for CD31 analysis performed on *n* = 3 IVD samples, were resuspended in 200 μL of PBS and incubated for 20 min at RT in the dark, in the presence of phycoerythrin‐ (PE‐) or fluorescein isothiocyanate‐ (FITC‐) conjugated antibody to rate the expression of the following mesenchymal stem cell (MSC) surface markers: CD34, CD45, CD73, CD90, CD105, CD133, CD15, CD14, CD19, CD31, HLA‐DR. To discriminate between viable and dead cells, 7‐aminoactinomycin D (7‐AAD, BD Bioscience) was added to each tube. Flow cytometric analysis was performed with FACScalibur flow cytometer Cell Quest software (BD Bioscience, version 8.0).

### 2.6 MTT Assay

Cell viability was assessed using 3‐(4,5‐dimethylthiazol‐2‐yl)‐2,5‐Diphenyltetrazolium Bromide (MTT) assay (Cayman Chemical), to indirectly estimate cell viability based on functional mitochondria. In detail, D‐IVD cells (1 × 10^4^ cells/cm^2^) were plated and cultured in 24‐well plates until 60% confluence was reached. Then, D‐IVD cells were treated following the scheme described above and subjected (or not) to PEMF for 4 h every day for five consecutive days at RT. At the end, culture media were replaced with 250 μL of RPMI added to 25 μL of MTT (5 mg/mL in D‐PBS). Plates were incubated for 4 h at 37°C, then media were removed, and formazan products were solubilized with 200 μL of 2‐propanol/formic acid (95:5, *by vol*). Cell viability was assessed by spectrophotometry at 570 nm absorbance using a microplate reader (BioTek Synergy H1).

### 2.7 Quantitative Real‐Time PCR Analysis (qRT‐PCR)

Total RNA extraction was performed following the TRI‐Reagent protocol (Life Technologies) and RNA concentration was quantified with the NanoDrop 1000 Spectrophotometer (ThermoFisher Scientific). Reverse Transcription to cDNA was performed using SensiFAST SYBR (Meridian Bioscience) loading 1 μg of RNA (A260/280 > 1.8) according to the manufacturer's instructions. For qRT‐PCR, reactions were performed with 1 μg of cDNA using specific forward and reverse primers previously tested to have an amplification efficiency range from 90% to 110% according to MIQE guidelines (Table 2), and the Titan HotTaq EvaGreen kit (BioAtlas) on the StepOnePlus (ThermoFisher Scientific). RPS18 expression was used as an endogenous control. Relative gene expression was determined using the 2^−ΔΔCt^ method. The melt curve was performed at the end of each Real‐Time PCR and checked by the analysis software to verify the amplicon specificity and the quality of the run.

**TABLE 2:** List of selected primer sequences (5′–3′ and 3′–5′) with relative melting temperature, amplicon length, and relative transcript.

Gene	Forward primer (5′–3′)	Reverse primer (5′–3′)	T_m_, °C	Amplicon length (bp)	Transcipts identified
*RPS18*	ATTAAGGGTGTGGGCCGAAG	TGGCTAGGACCTGGCTGTAT	61	205	NM_022551.3
*ACAN*	AAGGGCGAGTGGAATGATGT	CGTTTGTAGGTGGTGGCTGTG	61	80	NM_001135.4
*COL2A*	CTTCCCCCTCCTGCTCCAAG	CTGGGCAGCAAAGTTTCCAC	62	463	NM_033150.3
*IL6*	AGACAGCCACTCACCTCTTCAG	TTCTGCCAGTGCCTCTTTGCTG	61	132	NM_000600.5 (multiple transcript variants)
*ADAMTS4*	ACTGGTGGTGGCAGATGACA	TCACTGTTAGCAGGTAGCGCTTT	61	71	NM_005099.6 (multiple transcript variants)
*MMP1*	GGGGCTTTGATGTACCCTAGC	TGTCACACGCTTTTGGGGTTT	61	142	NM_002421.4
*MMP3*	CGGTTCCGCCTGTCTCAAG	CGCCAAAAGTGCCTGTCTT	60	206	NM_002422.5
*PPARG*	CTCGAGGACACCGGAGAGG	CACGGAGCTGATCCCAAAGT	61	179	NM_138712.5
*CALCA*	CCCAGAAGAGAGCCTGTGACA	CTTCACCACACCCCCTGATC	60	83	NM_001033953.3 (multiple transcript variants)
*ASIC4*	CCGCTTGCCCTGAGTTTAGA	CTTGGGTTTCGCATCCTCCT	60	180	NM_018674.6 (multiple transcript variants)

### 2.8 Reactive Oxygen Species (ROS) Detection

To evaluate ROS levels in D‐IVD cells, 2′,7′‐dichlorodihydrofluorescein diacetate (DCFDA) staining (Abcam, Cambridge, United Kingdom) was performed. Cells were seeded (2.5 × 10^4^/well) into a 96‐well plate and cultured for 24 h. Then, cells were incubated with 100 μL/well of DCFDA (25 μM) for 30 min at 37°C in the dark. After incubation, the DCFDA solution was removed and replaced with 100 μL/well of PBS 1X. Absorbance was read at Ex/Em = 485/535 nm with a plate reader (BioTek Synergy H1).

### 2.9 Cellular Glutathione (GSH) Quantification Assay

Cellular GSH was detected by using the Ellman's reagent (DNTB) (Thermo Fisher Scientific), a compound reacting with free sulfhydryl groups. D‐IVD cells were plated (1 × 10^4^/well) into a 96‐well plate and cultured for 72 h. After the indicated treatments, proteins were extracted with Mammalian Protein Extraction Reagent (M‐PER, Life Technologies) protocol, following the manufacturers' instructions. Sample supernatants were mixed with acid reagent and mixed fully, and then centrifuged at 4500 g for 10 min. Following this, DNTB (0.2 mM) solution and an equal volume of phosphate solution were added to each tube. The solution was mixed fully and stood for 5 min at room temperature and absorbance was measured at 412 nm on a microplate reader (BioTek Synergy H1).

### 2.10 IVD Nitric Oxide (NO) Production

NO released by D‐IVD cells in degenerative conditions was evaluated by measuring nitrite ion concentration in cell culture media using the Griess reagent assay as previously described [16]. At the end of the described treatments, sample supernatants were transferred to a 96‐well plate and incubated with an equal volume of sulfanilamide solution (1% sulphanilamide in 5% phosphoric acid) at room temperature for 10 min. Then, a solution of N‐1‐naphthyl ethylenediamine (NED, 0.1% in filtered water) was added to each well and incubated for 10 min at RT. Finally, optical density was measured at 530 nm using the microplate reader (BioTek Synergy H1).

### 2.11 Western Blot Analysis

D‐IVD cells were seeded into 75 cm^2^ flasks and cultured until they reached the appropriate confluence (about 80/90%). Cells were detached by Tryple Select (Gibco), centrifuged at 350 g for 10 min, and proteins were extracted by the M‐PER protocol, following manufacturers' instructions. Proteins were quantified by the Pierce BCA Assay Kit (Thermo Fisher Scientific). Protein lysates, 25 μg, were resolved in NuPAGE 10% Bis‐Tris Gel (Thermo Fisher Scientific) in the Mini Gel Tank and transferred onto nitrocellulose iBlot 2 Transfer Stacks using the iBlot 2 Dry Blotting System (Thermo Fisher Scientific). Nonspecific membrane sites were blocked with 5% BSA in Tris‐buffered saline with 0.1% Tween for 1 h at room temperature, and membranes were incubated separately with anti‐GAPDH, anti‐interleukin 6 (IL6), anti‐ADAMTS4, anti‐Peroxisome proliferator‐activated receptor gamma (PPARγ), anti‐Calcitonin Gene‐Related Peptide (CGRP), and anti‐Acid Sensing Ion Channel Subunit Family Member 4 (ASIC4), all purchased from Santa Cruz Biotechnology. After incubation with specific HRP‐conjugated secondary antibody (Life Technologies), protein bands were scanned with SuperSignal West Pico PLUS Chemiluminescent Substrate (ThermoFisher) and detected by iBright (ThermoFisher, Waltham, MA, USA). Densitometric analyses were performed using ImageJ.

### 2.12 Microglial Cell Immunofluorescence

The effect of PEMF on microglial cell activation was assessed by ASIC4 expression. Microglial cells were plated on a 96‐well plate at a density of 5 × 10^3^ cells/well and treated with IVD‐CM for 48 h. Next, cells were fixed with 4% PFA (15 min, RT). For fluorescence quenching, microglia were treated with 0.1 M glycine (10 min, RT) and permeabilized with 0.1% Triton X‐100 for 15 min. The primary antibody anti‐ASIC4 (1:500) was applied overnight at 4°C (Santa Cruz Biotechnology). Cells were then incubated with Alexa488 conjugated secondary antibody (1:1000, LifeTechnologies) at RT for 45 min, washed, and the nuclei were counterstained with DAPI (LifeTechnologies). Samples were acquired with an inverted Leica DMI6000B widefield microscope (Leica Microsystems, Wetzlar, Germany). Cells were counted in three microscopic fields in each well (3 wells/treatment), and the mean fluorescence intensity in the treatment groups was calculated as a percentage of the total number of cells and expressed compared to the CTRL group. Each treatment was performed in triplicate.

### 2.13 Statistical Analysis

Student's t‐tests were used with two tails for all statistical analyses and performed with GraphPad Prism software, version 10.4. Differences were considered statistically significant for *p* < 0.05. Values are expressed as mean ± SD of the independent experiments, run in triplicate.

## 3 Results

### 3.1 Immunophenotypic Profile of Human IVD Cells

**FIGURE 2:**
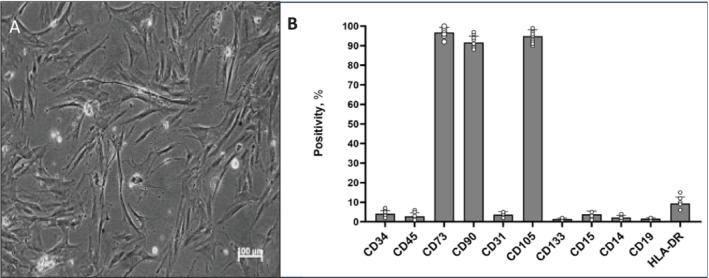
Characterization of human intervertebral disc degenerated cells. (A) Representative image of human D‐IVD cells cultured in basal conditions, after 3 weeks from IVD discectomy. (B) Immunophenotypic profile, obtained by cytofluorimetric analysis, of *n* = 10 D‐IVD cells (except for CD31 tested on *n* = 3 D‐IVD samples). Data are reported as percentage of positivity of the total number of cells tested. Scale bar 100 μm.

First, immunophenotypic analysis of degenerated IVD cells isolated from patients was performed (Figure 2A). We tested a panel of mesenchymal cell surface markers recognized by the International Society for Cellular Therapy (ISCT) as one of the minimal criteria for human MSC (hMSC) identification [17]. Flow cytometric analysis revealed that the D‐IVD population expressed a high percentage of positivity for CD73, CD90, and CD105. Conversely, D‐IVD cells were negative for CD34, CD45, CD133, CD31, CD15, CD14, CD19, and HLA‐DR (Figure 2B).

### 3.2 PEMF Do Not Alter IVD Cells Viability

**FIGURE 3:**
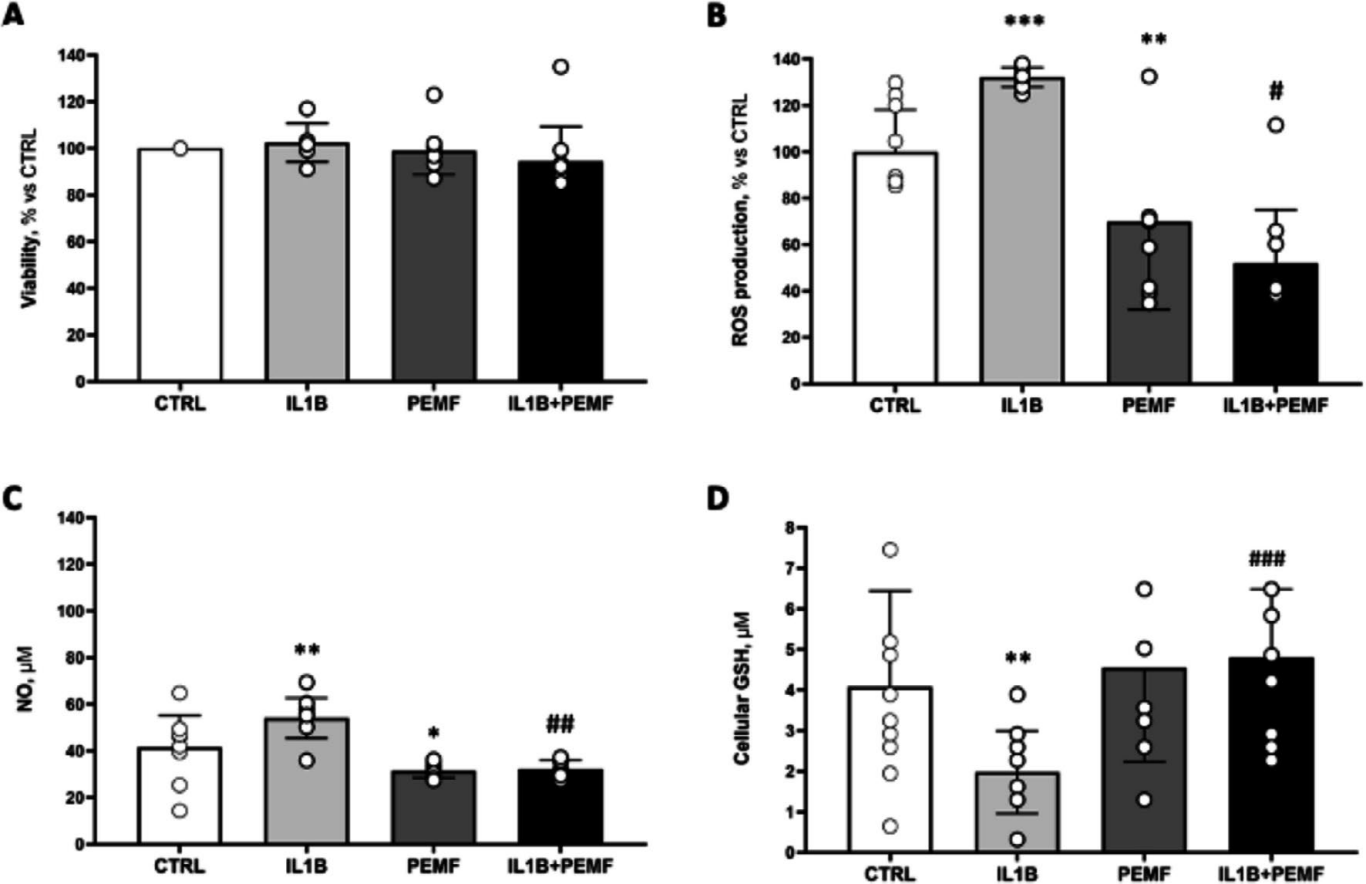
Effects of IL‐1β and PEMF on D‐IVD cells. (A) D‐IVD cell viability assessed by MTT assay after IL‐1β and PEMF treatment (B) Effect of IL‐1β, PEMF treatment, or the combination of the two on ROS production. (C) NO levels in degenerated IVD cells assessed by Griess assay, (D) Cellular GSH evaluated by Ellman's reagent. Data are the mean ± SD obtained from D‐IVD cells (*n* = 10). *IL‐1β, PEMF versus CTRL, #IL‐1β + PEMF versus IL‐1β. *,#*p* < 0.05; **,##*p* < 0.01; ***,###*p* < 0.001.

The assessment of PEMF influence on D‐IVD cell viability proved that no significant decrease in the number of viable cells occurs after PEMF exposition, not even in the presence of an inflammatory environment such as that simulated by the administration of IL‐1β (Figure 3A).

### 3.3 PEMF Mitigates Oxidative Stress in Degenerated IVD Cells

With the purpose of evaluating whether PEMF has effects on oxidative stress, we measured ROS production in D‐IVD‐CM. As shown in Figure 3B, the administration of IL‐1β determined an overproduction of ROS compared to CTRL, but this effect was strongly reduced by the contemporary exposition to PEMF (130% vs. 50%, *p* < 0.05). Interestingly, D‐IVD cells exposed to PEMF under basal condition exhibited a significant decrease in ROS compared to CTRL (100% vs. 70%, *p* < 0.01), suggesting its potential protective role against the oxidative stress naturally occurring in D‐IVD.

We then investigated NO released from D‐IVD cells under the same conditions described above, obtaining a comparable result. NO production was 1.3‐fold higher in the presence of IL‐1β than CTRL, with a significant reduction observed in D‐IVD cells subjected to PEMF, both at basal condition and after the administration of IL‐1β (Figure 3C).

Finally, to better explore the capability of PEMF therapy to blunt oxidative stress in degenerative conditions, we examined cellular levels of the major antioxidant glutathione (GSH). Interestingly, the administration of IL‐1β strongly decreased the amount of GSH compared with CTRL, as shown in Figure 3D. On the contrary, D‐IVD cells subjected to the PEMF and treated with IL‐1β showed a protective higher amount of GSH, as well as cells without IL‐1β (Figure 3D).

### 3.4 PEMF Blunts Neuro‐Inflammatory and Pain Marker Expression in Human IVD

**FIGURE 4:**
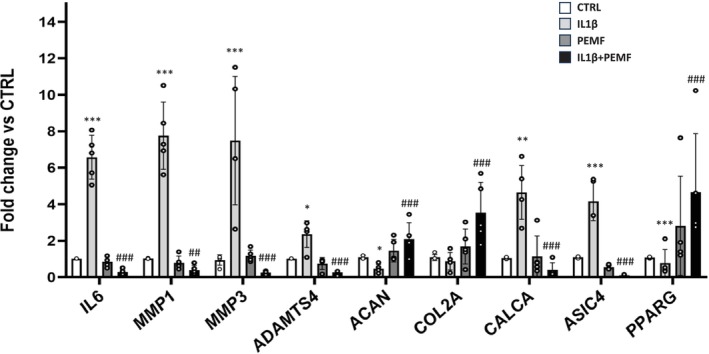
PEMF decreases inflammatory and pain markers and promotes matrix deposition in human degenerated IVD cells. The effect of PEMF both in untreated and IL‐1β‐stimulated cells for 4 h every day for 5 days on the mRNA expression of genes coding for *ACAN, COL2A, IL6, ADAMTS4, MMP1, MMP3*, *PPARG, CALCA, ASIC4* is shown. Histograms show the data as mean ± SD obtained from D‐IVD cells (*n* = 5), tested in triplicate. *ACAN*, aggrecan; *ASIC4*, acid sensing ion channel subunit family member 4; *CALCA*, calcitonin related polypeptide alpha; *COL2A*, collagen type II; *IL6*, interleukin 6; *MMP*, metalloproteinase; *PPARG*, proliferator‐activated receptor gamma. *IL‐1β, PEMF versus CTRL, ^#^IL‐1β + PEMF versus IL‐1β. *,#*p* < 0.05; **,##*p* < 0.01;***,###*p* < 0.001.

To investigate the effect of PEMF on the main degenerative events occurring during IDD, we first performed a gene expression screening of inflammatory markers and matrix components, and proteases (Figure 4). The obtained results revealed that PEMF, both with or without contemporary administration of IL‐1β, induced a robust reduction of the proinflammatory mediator *IL6* as well as extracellular matrix (ECM) degradative enzymes (metalloproteinases *MMP1*, *MMP3* and *ADAMTS4*), compared to CTRL D‐IVD cells.

Conversely, D‐IVD cells exposed to PEMF showed an upregulation of genes involved in matrix deposition (*ACAN*, *COL2A*), especially in the presence of the inflammatory stimulus.

Since IDD may eventually lead to the onset of pain, we also evaluated the gene expression of the pain markers *CALCA* and *ASIC4*. The addition of IL‐1β strongly increased *CALCA* expression, but this effect was significantly attenuated by the exposition to PEMF. Contrarily, a significant decrease of *ASIC4* was observed after PEMF treatment, either in untreated or IL‐1β‐stimulated IVD cells.

Finally, we examined *PPARG* levels, recognized as an endogenous anti‐inflammatory factor downregulated in many diseases. We found that D‐IVD cells stimulated with IL‐1β expressed low levels of *PPARG* compared to CTRL, whereas PEMF induced an enhanced expression of this protective factor, especially in the presence of the pro‐inflammatory cytokine (Figure 4).

**FIGURE 5:**
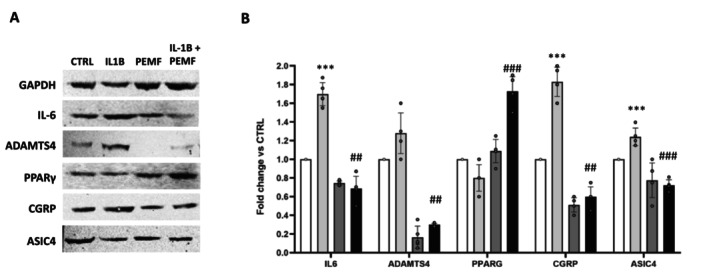
(A) Protein expression in degenerated IVD cells after treatment with IL‐1β and PEMF alone or in combination. (B) Densitometric analysis in ImageJ was performed to quantify protein content, expressed by Relative Unit versus GAPDH, used to normalize the results. Data were obtained from D‐IVD cells (*n* = 5) and represented by mean ± SD. *IL‐1β, PEMF versus CTRL, #IL‐1β + PEMF versus IL‐1β. **,##*p* < 0.01; ***,###*p* < 0.001.

Gene expression data were supported by protein analysis conducted by Western Blot on the major factors involved in the cascade of degradative events occurring during IDD. As previously reported, a down‐regulation of pro‐inflammatory mediators, matrix proteases, pain markers, and microglia activation was observed after D‐IVD cells exposition to a PEMF, as well as an up‐regulation of anti‐inflammatory and protective factors (Figure 5).

We also hypothesize that the IDD microenvironment could be exacerbated by microglial activation and that PEMF could restore the detrimental phenotype of immune cells. In this context, we co‐cultured microglial cells with D‐IVD‐CM, and the PEMF effects were evaluated.

**FIGURE 6:**
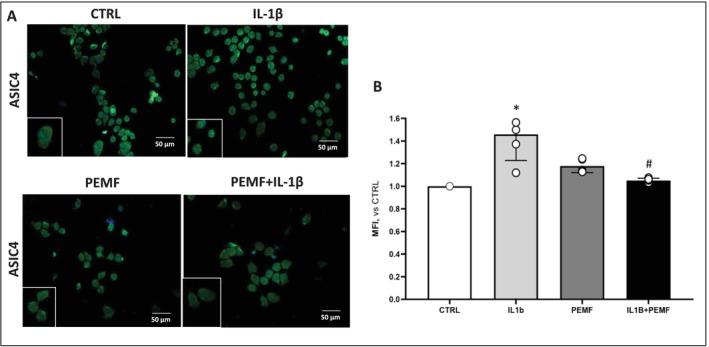
Effect of IVD microenvironment in neuroinflammatory cascade. (A) Immunofluorescence staining of ASIC4 in microglial cells after exposure to D‐IVD‐CM (scale bar, 50 μm). (B) Quantification of the mean intensity fluorescence (MFI) by ImageJ software. Data are mean ± SD of three independent experiments. *IL‐1β, PEMF versus CTRL, #IL‐1β + PEMF versus IL‐1β. *,#*p* < 0.05.

Results demonstrated that microglial cells increased the expression of the pro‐inflammatory marker ASIC4, following exposure to IL‐1β‐IVD‐CM. Interestingly, the treatment with PEMF + IL‐1β‐IVD‐CM significantly decreased the microglial expression of ASIC4 (Figure 6). By this way, we were able to assess the beneficial effect of PEMF therapy on neural cells recruited by the D‐IVD microenvironment during the IVD degeneration process.

## 4 Discussion

IDD is a common spine degenerative disease that affects a large percentage of people, often associated with LBP and thus representing an impactful socioeconomic problem. Current therapeutic approaches are limited to relieving symptomatic conditions. Still, unfortunately, conservative approaches often fail, as they cannot re‐establish the physiology of the IVD [3].

In the present study, we focused on the potential therapeutic efficiency of PEMFs, a non‐invasive approach able to exert multifunctional actions on tissues and organs dealing with damage and homeostasis maintenance.

PEMFs exert a biological tissue and cellular response directly inducing electrical currents without determining mechanical agitation [18]. PEMFs were introduced in the 1977s by Bassett and colleagues as a treatment for non‐operative salvage of surgically resistant pseudarthroses [19]. After the approval of the FDA in 1979, PEMFs have been applied in clinics for the treatment of orthopedic indications, such as bone formation, non‐unions, osteoarthritis, and more.

It has been demonstrated that PEMFs can influence tissue in a dual mode, both resulting in a forced shift of ions or charged particles, as: (i) the applied magnetic field produces a force on tissue‐forming molecules strictly dependent on tissue‐magnetic reactive features; (ii) the electrical field generated exerts a force on tissue ions.

PEMF therapy is generally considered a non‐invasive procedure that presents many advantages, including safety, lack of toxicity for non‐cancerous cells, and the possibility of being combined with other therapies to improve bone disease and lipid metabolism, without any significant collateral effects [20]. It has been demonstrated that PEMF can interfere with many molecular pathways involved in cell proliferation, apoptosis, cell differentiation, and other biological functions, including osteogenesis and adipogenesis. However, the relationship between PEMF exposure and adipogenesis is not completely understood, and the potential adipogenic side effects have not been fully investigated. It has been demonstrated a correlation between this therapy and the expression of the main proteins involved in adipocyte differentiation and development, including PPAR‐γ, Wnt, C/EBPα. Yin and colleagues showed that PEMF has a role in stimulating proliferation and differentiation of Adipose‐derived Stem Cells (hASCs), as well as in inducing the expression of proteins related to osteogenic differentiation [21]. These data point out the necessity to deeper investigate if PEMF therapy plays a role in metabolic disorders related to adipogenesis, for example by promoting an unbalanced proliferation of adipocytes, by worsening an already existing adiposity condition as well as by stimulating the recovery of tissues indirectly involved in lipid metabolism.

Here, we tested the potential therapeutic effects of PEMF on the main pathological features of IDD: the overproduction of pro‐inflammatory mediators strictly connected with the onset of oxidative stress, the progressive degradation of ECM, as well as the increased activation of neuroglia.

PEMF has already been characterized to be effective in reducing inflammatory phenomena while promoting cell proliferation, the release of several growth factors that contribute to bone tissue neoformation, osteogenic differentiation of mesenchymal stem cells, and the production of collagen and extracellular matrix glycoproteins.

Evidence from literature already reported that PEMF can significantly decrease pro‐inflammatory cytokine expression in human and bovine IVD cells in vitro [22, 23] as well as in human chondrocytes, fibroblasts, tendon cells, and osteoblasts [24, 25], showing promising anti‐inflammatory properties.

Several works reported that PEMF exerts its therapeutic role via regulation of cell proliferation, differentiation, and maturation, especially in the treatment of bone diseases [26, 27, 28]. Xu et al. reported that PEMF at a frequency of 75 Hz (the same frequency used in this work), compared with other frequency parameters, was more effective on adipose mesenchymal stem cell‐derived exosomes in suppressing the IL‐1β‐induced chondrocyte inflammation and extracellular matrix degeneration [29].

Furthermore, Fassina et al. showed that PEMF (intensity, 2 mT; frequency, 75 Hz; amplitude of the induced electric tension, 5 mV; pulse duration, 1.3 ms) significantly enhanced human osteoblasts proliferation and synthesis of the main constituents of physiological bone matrix (e.g., type‐I collagen, decorin, osteopontin, osteocalcin, type‐III collagen) [30].

The beneficial effects of exposition to 2 mT, 75 Hz PEMF were also investigated in other cell types, such as human neuroblastoma cells: Falone et al. showed an increase of superoxide dismutase in drug‐resistant SK‐N‐BE cells and a reduction of ROS production, resulting in an overall improvement of the antioxidant response [31].

Therefore, here we decided to expose D‐IVD cells to PEMF using the same parameters. D‐IVD were isolated from patients with IDD, and preliminary FACS analyses were performed to characterize the IVD cells immunoprofile. According to the consensus definition of MSCs developed by the International Society for Cellular Therapy (ISCT), our primary D‐IVD cells showed the expression of CD73, CD90, and CD105, and a lack of expression of CD34, CD45, CD14, CD19, and HLA‐DR [32]. However, it should be considered that recent studies reported that cell surface markers are not sufficient to definitively distinguish MSCs from fibroblasts [33].

The protocol of PEMF treatment (4 h daily for 5 days) was assessed based on already existing and validated protocols in the literature showing the beneficial effect of this strategy. Miller et al. for example, showed that NP cells treated with IL‐1β upregulate pro‐inflammatory genes (MMPs, NF‐kB, IL6) that diminished with PEMF treatment after 4 days, while by day 7, the response of IVD cells treated with PEMF and IL‐1α did not significantly differ from that of cells treated with IL‐1α alone [23].

Also imaging experiments demonstrated that the effect of IL‐1α on IL6 expression could be significantly inhibited by PEMF treatment in a time‐dependent manner (early as 2 h of stimulus initiation) [34].

As a further control, before PEMF exposition, we pre‐treated D‐IVD cells with IL‐1β to mimic the typical exacerbated inflammatory microenvironment present in degenerative conditions. Belonging to the IL‐1 family, IL‐1β is a pro‐inflammatory cytokine that plays a crucial role in inflammation‐related diseases. Notably, a previous study showed that the expression of IL‐1β was higher in degenerative IVD cells compared to non‐degenerative IVD cells [35], whereas its suppression has been demonstrated to prevent IDD [36, 37]. In addition, several studies have reported that IL‐1β is closely associated with the main pathological IDD processes examined in our work [38].

We initially tested the effects of PEMF on oxidative stress, since many studies demonstrated its involvement in IDD pathogenesis. Suzuki and colleagues observed that ROS levels and NO release significantly increased in IVD along with the progression of IDD [39]. It has been reported that in degenerated IVD, the balance between ROS production and removal is impaired, resulting in oxidative damage to the ECM, which progressively destroys the mechanical properties of the IVD [40].

Mathy‐Hartert et al. have found that bovine chondrocytes treated with IL‐1β significantly reduced the production of superoxide dismutase and catalase, enzymes belonging to the antioxidant defense system [41, 42]. Interestingly, recent evidence demonstrated that PEMF exposure reduced intracellular ROS levels in human osteoblasts, via induction of O_2_− and H_2_O_2_ and increase of GPX3, SOD2, CAT, and GSR mRNA, protein, and enzyme activity levels [43].

In agreement with the above‐mentioned studies, here we obtained similar results on human D‐IVD cells isolated from patients who underwent discectomy: the IL‐1β‐induced oxidative stress was attenuated by exposition to PEMF, suggesting the potential cytoprotective role of the electromagnetic treatment against the detrimental effects of oxidative damage observed in degenerative conditions.

Many studies conducted on disc samples demonstrated higher levels of pro‐inflammatory factors expressed by degenerating IVDs compared to healthy IVDs [44, 45]. Moreover, consequent to IDD progression, the levels of IL‐1α, IL‐1β, IL6, and TNF‐α increased significantly [37, 46, 47]. Notably, several works demonstrated that the onset of this inflammatory microenvironment promotes matrix degradation, apoptosis, and senescence, resulting in the worsening of overall degenerative conditions [3, 38].

As reported by Fang and colleagues, during IDD, IL‐1β promotes the production of matrix metalloproteinases (MMP1, MMP3, MMP13) and ADAMTS4, widely recognized as the first enzymes involved in IDD [48]. In addition, Zang et al. reported that IL‐1β reduced the expression of aggrecan and type‐II collagen, the main components of the IVD matrix [49].

Here, for a deeper assessment of PEMF efficacy, we performed gene and protein expression analysis before and after exposition to PEMF: D‐IVD cells pre‐treated with IL‐1β expressed high levels of the pro‐inflammatory cytokine IL6 and ECM proteases (MMPs and ADAMTS4), whereas low levels of matrix components (ACAN, COL2A) were found. However, consistent with recent works exploring in vitro and in vivo the beneficial effects of PEMF on inflammation and ECM catabolism [22, 50], we obtained a significant improvement of the inflammatory status after exposition to PEMF, as well as the increasing deposition of matrix.

Moreover, in addition to highlighting the anti‐inflammatory and regenerative effects of the treatment, our findings also showed an interesting potential analgesic property through the analysis of CGRP. CGRP is a neuropeptide responsible for pain perception expressed primarily on sensory nerve fibers [51]. Recently, both in mouse and human samples, Sun and colleagues demonstrated high levels of CGRP in degenerated IVDs, as well as its involvement in inflammation, ECM degradation, and apoptosis through the activation of NF‐kB and MAPK pathways in vitro [52]. Our data revealed a consistent decrease of CGRP levels after PEMF, suggesting a supplementary beneficial effect of the treatment, especially considering the current lack of effective therapies to alleviate LBP symptoms.

Collectively, our observations are in line with the literature and support the hypothesis that PEMF may be considered a valid approach to counteract the detrimental effects of disc degeneration and consequently prevent the onset of pain.

To better understand the role of PEMF in degenerative conditions, we also focused on its effect relative to immune cell contribution.

Healthy IVDs are considered immune‐privileged organs, protected from immune cell infiltration and immune response [53]. However, it has been reported that infiltrating immune cells, including T‐regs, macrophages, neutrophils, microglia, and T cells, are implicated in IDD progression [54, 55].

Evidence from literature in fact shows that human D‐IVD attracts and hosts a population of resident macrophages/microglial cells [56], which are activated to provide an overproduction of pro‐inflammatory cytokines and neurotrophins, which in turn accelerate the degenerative processes [57]. In line with these reports, we recently demonstrated that degenerative conditions influence microglia in terms of enhanced activation, proliferation, and chemotaxis, confirming its involvement in exacerbating neuroinflammation during IDD [16]. Culturing microglia with D‐IVD‐CM determines an upregulation of pro‐inflammatory mediators such as IL6 and TNFα, able to exacerbate the discogenic pain during IDD, with a positive significant correlation, aggravating the cascade of pathological events [16, 56].

These findings highlighted the necessity to develop new therapeutic approaches against the immune component of degenerative IVD, and microglia seems to represent a promising target. To date, growing evidence demonstrated the anti‐neuroinflammatory actions of PEMF and the underlying signaling pathways in cerebral ischemic conditions and neurodegenerative brain diseases [58, 59, 60]; whereas, in the context of IDD, PEMF functions remain to be explored. On this basis, neuroinflammatory markers were evaluated before and after treatment, both in degenerated IVD cells and in N9 microglial cells pre‐treated with D‐IVD‐CM. Notably, PEMF treatment showed inhibitory effects on the ASIC4 marker, suggesting a positive role in counteracting microglia activation and proliferation and consequently mitigating the detrimental crosstalk between inflammation and neuroinflammation.

According to our previously published works, we are actually conducting further in vitro experiments to better explore the molecular mechanisms behind microglia activation in IDD and how PEMF could be effective in reducing microglia proliferation, in order to strengthen the preliminary data obtained in this study. Moreover, we aim to investigate the protective role of the treatment focusing on other immune regulatory cells infiltrating IVD tissues responsible for the pathology, including macrophages and astrocytes. Taken together, the results of this study provide novel evidence that PEMF might potentially counteract the pathological events occurring inside the degenerating IVD and, simultaneously, suppress the local neuroinflammatory microenvironment. Thus, our findings support the hypothesis that PEMF may represent a valid treatment for IDD and its related chronic back pain, with a great impact on public health and healthcare costs.

Of relevance, with the advancement of space exploration, aerospace medicine is rapidly expanding its horizons, looking for minimally invasive countermeasures applicable on board to limit the detrimental effect of space conditions on the musculoskeletal system.

During space missions, the physical and biological adaptive changes occurring due to the decrease or lack of gravity lead to the loss of mechanical stimulation of cells and tissues, with consequent significant impairments of body physiology, especially the musculoskeletal system. In this specific context, the absence of gravity and diurnal changes in height and hydration to which the spine is subjected on Earth determines an unbalance in IVD homeostasis and therefore the activation of degenerative programs [11, 61].

Bailey JF and colleagues reported that, among astronauts, the incidence of IDD is approximately quadrupled due to the peculiar environmental conditions to which pilots are subjected during spaceflights [62]. Several studies demonstrated that microgravity plays a crucial role in IVD pathophysiology, as well as in the onset of associated LBP [11]. Moreover, several works performed on cells exposed to real or simulated microgravity conditions have reported important changes in IVD structure and functionality [63]. This evidence suggests the necessity to develop effective countermeasures to counteract the detrimental effects of microgravity on the health and performance of astronauts.

In this context, our research group recently participated in the *Virtute‐1* mission by Virgin Galactic, the first commercial suborbital flight for research purposes with an Italian Crew onboard the Spaceship Unity. By this experience, we could test the early effect of microgravity on cellular and molecular pathways in D‐IVD cells to detect epigenetic, transcriptomic, and metabolomic modifications occurring in a few minutes and potentially responsible for the IVD response to the space environment. The analysis of these promising experiments is ongoing, and they will object to the next manuscript in preparation.

Before concluding, we here introduce a study limitation to be considered, as the choice of applying PEMF treatment at room temperature, since the incubator we disposed of was not suitable to host the bioreactor because of its size and the dimension of the electrical wire for the connection of the power. We are conscious that experiments performed at room temperature instead of 37°C in the incubator might expose cells to some interfering factors. For this reason, we propaedeutically performed some experiments in order to set up the experimental cell condition and we strictly checked that the main parameters did not fluctuate and remained as much as possible within the established ranges, both before and during the treatment, and we used a control group exposed to the same environmental conditions, except for PEMF treatment. Results demonstrated that the environmental conditions of our lab did not influence the parameters tested.

However, the future miniaturization of the bioreactor is needed to improve the experimental settings and confirm the results obtained in this work.

## 5 Conclusion

In conclusion, considering that LBP is one of the main causes of disability in the world population with a high incidence also among pilots and astronauts, PEMF could be investigated as a potential therapeutic and preventive approach protecting human IVD from neuroinflammatory and detrimental microenvironments, inhibiting pro‐inflammation, pain mediators, and degenerative processes. This approach deserves important consideration also in the view of improving patients' quality of life and reducing both civilian and military healthcare costs.

## Author Contributions

Conceptualization: L.G.; L.F.; G.M.; S.E.N. Methodology: L.G.; L.B.; S.P.; C.C.; S.E.N. Software: L.G.; L.B.; S.E.N. Validation: S.P.; L.F.; G.A.A.; M.M.; E.G.; L.R.; E.B.; L.F. Formal analysis: L.G.; L.B.; R.C.; C.C.; G.M.; S.E.N. Investigation: L.G.; L.B.; L.F.; G.A.A.; E.B.; G.M.; S.E.N. Resources: M.P.; S.B.; M.D.R.; G.F.; G.A.A.; E.B.; M.L.; G.M.; S.E.N. Data curation: L.G.; M.D.R.; C.C.; C.G.; L.F.; G.M.; S.E.N. Writing‐original draft preparation: L.G.; L.B.; G.M.; S.E.N. Writing‐review and editing: S.P.; M.P.; S.B.; C.F.; M.D.R.; G.F.; L.F.; R.C.; C.C.; C.G.; G.A.A.; M.M.; E.G.; E.B.; L.F.; L.R.; M.L. Visualization: L.G.; L.B.; S.E.N. Supervision: S.P.; M.P.; S.B.; M.D.R.; G.F.; L.F.; G.A.A.; M.M.; E.G.; E.B.; L.F.; L.R.; M.L. Project administration: L.G.; M.L.; G.M.; S.E.N. Funding acquisition: L.G.; L.F.; M.L.; G.M.; S.E.N. All authors read and approved the final paper.

## Conflicts of Interest

The authors declare no conflicts of interest.
